# Coronary Artery Fistulae Discovered during Presentation of a Patient Having Heart Failure due to Severe Aortic Stenosis

**DOI:** 10.1155/2014/213673

**Published:** 2014-11-16

**Authors:** Abdulrahman Khalifa Alammar

**Affiliations:** Department of Medicine, College of Medicine, Qassim University, Qassim, Saudi Arabia

## Abstract

*Introduction*. Coronary artery fistulae (CAF) are rare defects with abnormal communication between a coronary artery with either a cardiac chamber or another vascular structure, bypassing the myocardial capillary network. We report a rare multiple arterial coronary fistulae with drainage to the right pulmonary artery. *Case Presentation*. A 56-year-old male was brought to our hospital for work-up of severe aortic stenosis. Further evaluation revealed multiple CAF with abnormal drainage to the right pulmonary artery. He was discharged after aortic valve replacement and closure of the coronary fistula. *Conclusion*. This case demonstrates that patients with complex CAF and drainage to the right pulmonary artery can remain asymptomatic and diagnosed accidentally during cardiac imaging, presenting particular challenges in both medical and surgical treatment.

## 1. Introduction

Coronary artery fistulae are communications between a coronary artery with either a chamber of the heart or any segment of the systemic or pulmonary circulation; it has a rare occurrence seen in only 0.1-0.2% of coronary angiography (CAG) [1]. In the first two decades of its occurrence, CAF usually causes no symptoms or complications but can occur in older age [2]. Most frequent complications include steal syndrome, thrombosis, embolism, rupture, cardiac failure, and arrhythmias [3, 4]. We describe a case of a rare type of multiple CAF in a 56-year-old male who presented with symptoms of heart failure due to severe aortic stenosis.

## 2. Case Presentation

**Figure 1:**
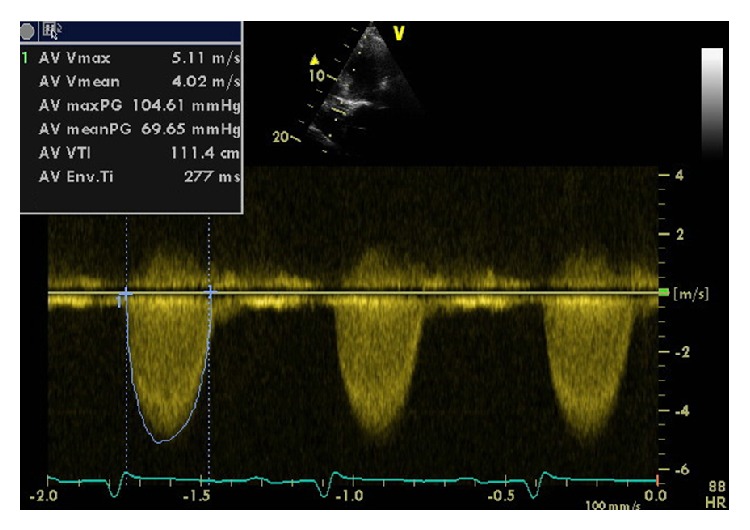
Echocardiography, continuous-wave spectral Doppler across the aortic valve showed severe aortic stenosis with mean gradient 70 mmHg and peak gradient 105 mmHg.

**Figure 2:**
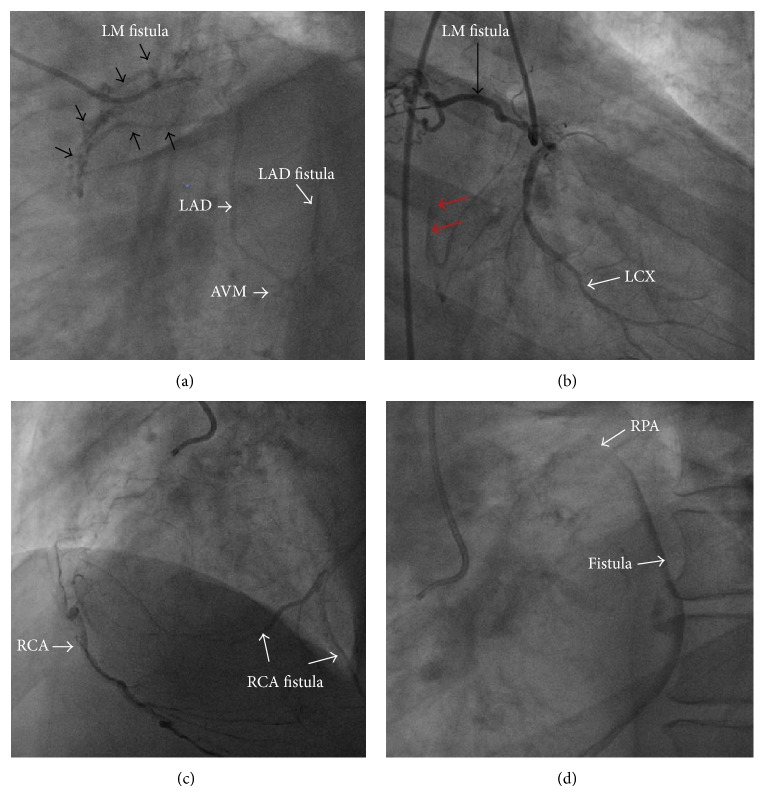
(a) Still-frame image of late filling of coronary angiogram to left system with selective engagment of the LAD showed LM fistula (black arrows) which join the fistuous vessel of the other coronary artery and then drainage to the right pulmonary artery (RPA). (b) Still-frame image of cornonary angiogram to left system with selective engagement of the LCX and LM fistula (black arrow) fistolous vessel fills from the LCX (red arrow). (c) Still-frame image of cornonary angiogram of the RCA shows the fistolous vessel fills from RCA. (d) The same injection with late filling shows the fistula drainge to RPA. LAD: left anterior descending. LCX: left cercumflex. LM: left main. RAC: right coronary artery. RPA:right pulmonary artery.

**Figure 3:**
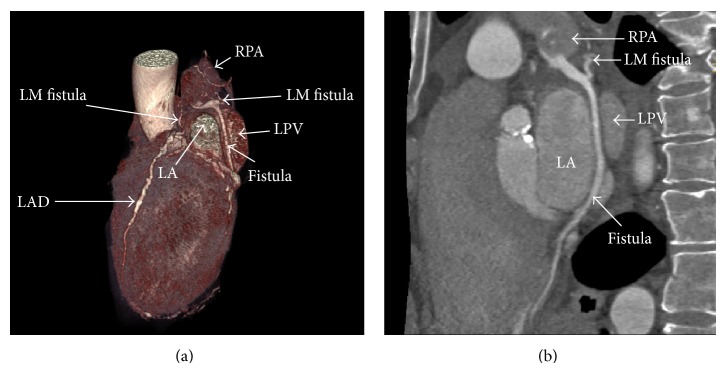
(a) Volume rendering CTA showed LM fistula joining the fistulous vessel of the other coronary arteries draining into the RPA. (b) Curved MPR showed the insertion point of the fistula at RPA. LAD: left anterior descending artery. LPV: left pulmonary vein. LA: left atrium.

A 56-year-old Saudi male referred to our hospital for evaluation of dyspnea New York Heart Association (NYHA) class III started six months before and had progressed to NYHA class IV two weeks prior to admission. The patient denied having any chest pain, palpitation, or dizziness. He had no risk factors for coronary artery disease (CAD). His electrocardiogram (ECG) showed sinus rhythm with signs of left ventricle hypertrophy LVH. Echocardiography showed global hypokinesia and severely reduced left ventricle systolic function with an ejection fraction of 25%, severe aortic stenosis (aortic valve area = 0.6 cm^2^, and mean gradient = 70 mmHg) pulmonary to systemic blood flow ratio *Q*_*p*_/*Q*_*s*_ = 1.12 (Figure 1). After stabilizing the heart failure, an invasive coronary angiography was performed and showed nonsignificant coronary artery stenosis; but multiple coronary arterial-venous malformation (AVM) and CAF with a connection to a vascular structure cannot be clearly delineated (Figure 2), so computed tomography angiography (CTA) was undertaken for further anatomical clarification and better three-dimensional (3D) orientation. CTA confirmed the coronary AVM with multiple coronary artery fistulae connected to the right pulmonary artery (RPA) and left main (LM) fistula coursing between ascending aorta and the left atrium (LA), while the other fistula ran between the LA and a single left pulmonary vein (SLPV) (Figure 3).

The case was presented in a combined cardiology and cardiac surgery meeting, and the consensus was that the patient should undergo aortic valve replacement, with intervention to coronary fistulae and closure of fistulae. Subsequently the patient underwent aortic valve replacement with mechanical valve and closure of fistulae. Perioperative course remained uneventful and he discharged on the fifth postoperative day. On two-month follow-up the patient was asymptomatic. Postoperative echocardiography showed improved left ventricular function (EF = 35%), and a well-seated and normal prosthetic aortic valve.

## 3. Discussion

A coronary fistula is abnormal communication between the coronary artery and either the cardiac chamber or another vascular structure. The majority of these defects are congenital, although they may occasionally be acquired after trauma, percutaneous coronary intervention, and cardiac surgery [5–8]. The incidence is about 0.1-0.2% and comprises about 14% of all congenital coronary anomalies1; it can be solitary 72% or multiple 28% [9]. Symptoms usually occur after the second decade of life, but with large-size fistulae symptoms may start earlier. The treatment options for coronary arterial fistulae include either surgery or catheter closure. Surgery involves internal closure within the receiving chamber or ligation from within the aneurysm and is associated with a low morbidity and mortality rate, range 0–6% [10, 11]. Catheter closure of fistulae is now considered to be an effective and safe alternative to surgery [12–14]. Considering our patient, with a plan for AVR, the final decision was therefore to carry out a combined surgical procedure involving fistula closer and replacement of the aortic valve. With this case we showed that CAF can be diagnosed by cardiac catheterization, but CTA can provide additional information about the number, course, and three-dimensional orientation that may have an impact. On the other hand, the decision of CAF closure was affected by the presence of significant valvular disease needing surgical intervention.

## 4. Conclusion

We have described a man presenting symptomatic severe aortic stenosis and incidentally also found to have multiple coronary artery fistulae drainage to the right pulmonary artery; intervention was carried out on the fistulae.

## Abbreviations

AVM: Arterial-venous malformation
CAD: Coronary artery disease
CAF: Coronary artery fistula
CAG: Coronary angiography
CTA: Computed tomography angiography
LA: Left atrium
LM: Left main
RPA: Right pulmonary artery.
